# Serum Vitamin D Metabolites and CXCL10 Concentrations Associate With Survival in Dogs With Immune Mediated Disease

**DOI:** 10.3389/fvets.2019.00247

**Published:** 2019-07-30

**Authors:** Phillip J. Mick, Seth A. Peng, John P. Loftus

**Affiliations:** Department of Clinical Sciences, Cornell University, Ithaca, NY, United States

**Keywords:** vitamin D, cytokines, calcitriol, autoimmunity, canine

## Abstract

**Background:** Low vitamin D increases the risk of immune-mediated disease (IMD) in human beings and rodent models. Vitamin D metabolites, particularly 1,25(OH)2D3, modulate gene expression of immune cells and may attenuate immune pathways that drive IMD.

**Hypothesis/objectives:** Our primary hypothesis was that serum 25(OH)D3 and 1,25(OH)2D3, are reduced in patients with IMD and associate with poorer outcomes. We secondarily hypothesized serum 24,25(OH)2D3 would not be associated with disease or outcome. We also measured serum CXCL10 concentrations to determine if an increase occurs in dogs with IMD and in association with poorer survival.

**Animals:** We enrolled dogs diagnosed with IMD (*n* = 29) and healthy control dogs (*n* = 9) in the study with informed client consent.

**Methods:** Serum was collected and stored at −80°C until analyses. Serum vitamin D metabolites were measured by LC-MS/MS by an accredited laboratory. A commercially available canine-specific ELISA kit measured serum CXCL10.

**Results:** Serum 25(OH)D3 and 1,25(OH)2D3 were significantly reduced in dogs (*n* = 25) with IMD. Serum CXCL10 concentrations undetectable in all controls, and were 30 times higher overall in IMD dogs (*n* = 25; *P* = 0.004). CXL10 was, however, undetectable in 40% of dogs with IMD. Of the 60% of IMD dogs with increased CXCL10 concentrations, 5/25 had concentrations at the upper limit of quantification. The survival of those five dogs was significantly (*P* = 0.049) shorter (72 days) than all other dogs with IMD with measured CXCL10 concentrations. The median survival time (MST) for dogs with 25(OH)D3 concentrations ≤ the median was 106 days, while dogs with concentrations of 25(OH)D3 > the median did not achieve an MST.

**Conclusions and clinical importance:** Serum 25(OH)D3 and 1,25(OH)2D3, but not 24,25(OH)2D3 levels are reduced dogs with IMD. Vitamin D metabolites and CXCL10 may be useful prognostic markers and may be targets for adjunct therapy in canine IMD. These data support the future investigation of vitamin D analogs in the treatment of canine IMD.

## Introduction

Several immune-mediated diseases (IMD) afflict dogs and may have correlates to specific autoimmune conditions in human beings. Particularly true in human medicine, biomarkers refine the diagnosis, inform the prognosis and monitor treatment of immune-mediated or autoimmune diseases. One such biomarker is vitamin D or its metabolites. While Vitamin D is essential for maintaining blood calcium concentrations, its role in immune regulation is increasingly recognized. Vitamin D, or cholecalciferol, is a fat-soluble vitamin metabolized to various biologic forms. Exposure to ultraviolet light synthesizes vitamin D in some species (e.g., human beings), but the dietary acquisition of cholecalciferol is essential for dogs (1, 2). After ingestion, the liver converts dietary forms of vitamin D to 25-hydroxycholecalciferol [25(OH)D3, calcidiol]. This isoform has modest biologic activity compared to 1,25(OH)2D3 (calcitriol), which is synthesized primarily in the kidney (3). Calcitriol activates the vitamin D receptor (VDR), modulating gene expression. Many VDR target genes encode proteins essential for maintaining immune homeostasis.

Various immune cells metabolize forms of vitamin D and many express VDR, emphasizing an essential role for vitamin D metabolism in the immune system (4). Calcitriol exerts the greatest biologic effects on the immune system and calcitriol analogs, or VDR agonists, demonstrably modulate immune cell activity. Activated T lymphocytes (and possibly B lymphocytes) express CYP27B1, which synthesizes 1,25(OH)2D3 (5, 6). Calcitriol drives T regulatory (Treg) cell differentiation and maturation (7–9). IL-2 and IFNγ synthesis is reduced by 1,25(OH)2D3 mediated VDR activation (10, 11). VDR activation also reduces gene expression of CXCL10, an IFN-induced leukocyte chemokine (12, 13). In addition to chemotactic activity, CXCL10 initiates and maintains pathologic Th1 biased responses (14). These Th1 responses are associated with autoimmunity, but not generic inflammatory responses (15). Reduced CXCL10 induction by VDR ligands may partially explain attenuated autoimmunity from treatment with calcitriol or its analogs. Overall, 1,25(OH)2D improves immune homeostasis, partially by reducing CXCL10, and augmenting immunologic tolerance.

Autoimmune conditions that associate epidemiologically with lower vitamin D concentrations include multiple sclerosis (MS), systemic lupus erythematosus, and rheumatoid arthritis (16–22). Experimental autoimmune encephalitis (EAE), the mouse model of MS, is prevented by VDR agonists (8, 16, 23). VDR agonists also reduced immunosuppressive drug doses required in EAE. In cell culture models, VDR agonists reduce CXCL10 secretion (24–26). These data prompted us to investigate serum vitamin D metabolite and CXCL10 concentrations in dogs with naturally occurring IMD and predicate our hypothesis. We hypothesized that reduced serum vitamin D metabolite, and increased CXCL10 concentrations, would be measured and negatively correlate with survival in dogs with IMD.

## Materials and Methods

### Animals

Patients were recruited from the Cornell University Hospital for Animals (CUHA) with informed client consent. The Cornell University Institutional Animal Care and Use Committee approved this protocol.

#### IMD Group

We preliminarily enrolled patients with suspected IMD pending subsequent diagnostic testing. Minimal diagnostic testing for inclusion included complete blood count, chemistry profile, urinalysis, thoracic, and abdominal imaging. Disease-specific tests were performed based on the manifestation of disease and clinician discretion, which included Coomb's testing, antinuclear antibody testing, coagulation testing, urine culture, joint cytology, and culture. We excluded patients that were determined to have a concurrent systemic disease (e.g., endocrinopathy, renal disease, and liver disease). We also excluded patients with either an alternative diagnosis or where the diagnosis of IMD was determined to be secondary (e.g., precipitated by infectious disease, neoplasia, and drug exposure). Patients were excluded if they received immunomodulatory drugs for >24 h prior to blood collection. One author (JPL) assured appropriate diagnosis and evaluated all medical records. Survival data were determined from the patients' medical records.

#### CON Group

We identified healthy control dogs from the CUHA based on (1) an unremarkable physical exam by a veterinarian, (2) a history lacking indication of disease, and (3) normal CBC and chemistry profile.

We acquired blood samples by venipuncture of peripheral veins or through sampling catheters. The phlebotomist chose the site at their discretion. In most cases, blood was collected contemporaneously with diagnostic blood samples. Whole blood was collected into additive-free 4 ml glass tubes. After the blood clotted, tubes were centrifuged at 676 × g for 10 min at room temperature. Serum was collected with transfer pipettes and stored in 2 ml plastic tubes at −80°C until analyses.

### Vitamin D Analyses

Serum concentrations of vitamin D metabolites [25(OH)D3, 1,25(OH)_2_D3, and 24,25(OH)_2_D3] were measured by liquid chromatography tandem mass spectrometry (LC/MS/MS) at an accredited laboratory (Heartland Assays, Ames, IA).

### CXCL10 ELISA

Serum CXCL10 was measured by a commercially available Dog CXCL10/IP10 ELISA Kit (LSBio, Seattle, WA) per manufacturer instructions. The manufacturers report no interference or cross reactivity with CXCL10 analogs. We performed the assay on the provided pre-coated 96-well Strip Plate. The standard stock solution (2,000 pg/mL) was freshly diluted with sample diluent as instructed. Sample diluent alone provided the zero standard (0 pg/mL). The standard dilution series was plated in duplicate. All samples were analyzed by a single kit during one assay procedure. Neat serum (100 μL) was plated in duplicate from each patient and incubated at 37°C for 1 h. We conducted detection and wash steps per the manufacturer. After incubation, the plate was washed five times with 1x Wash Buffer and blotted after the final wash. TMB Substrate solution (90 μL) was added to each well and incubated for 10 min at 37°C. Stop Solution (50 μL) was added to each well, and immediately measured the optical density with an Epoch microplate spectrophotometer (BioTek, Winooski, VT) at 450 nm. We assigned a concentration of 6.7 pg/ml to results below the sensitivity of this assay (6.8 pg/ml).

### Statistical Analyses

We visually assessed the data for normality, and non-gaussian distributions were present for all data. We depict the data with medians and individual data points. Two-way ANOVA with Bonferroni correction compared serum metabolite concentrations between IMD and CON groups. The Kruskal–Wallis test with Dunn's multiple comparisons compared 25(OH)D3 and 1,25(OH)2D3 concentrations between disease categories. We reported adjusted *P*-values for multiple comparisons. We compared CXCL10 concentrations between the IMD and CON groups, and patient weight and ages between patients with high vs. low 25(OH)D3, by the Mann-Whitney test. The Log-rank (Mantel-Cox), and Gehan-Breslow-Wilcoxon tests compared survival curves, and we report the higher *P*-value. Patients were censored at the last date that indicated the patient was alive in the medical record at the time of manuscript preparation. Statistical significance was set at *P* < 0.05. We conducted statistical analyses with Prism 7.0 or higher software (GraphPad, San Diego, CA).

## Results

### Patient Demographics and Samples

We initially recruited 33 dogs with suspected IMD into the study. Cases not meeting study criteria as defined above were excluded, resulting in 29 dogs in the IMD group. Dogs in the IMD group ranged in age from 1 to 11 years of age at the time of diagnosis. The weight of affected dogs ranged from 3.7 to 54.7 (median 27.0) kg at the time of diagnosis. Dogs' sexes were as follows: castrated male (*n* = 13), spayed female (*n* = 12), intact male (*n* = 3), and intact female (*n* = 1). The following breeds were represented: mixed breed (*n* = 6), Labrador retriever (*n* = 3), German shepherd dog (*n* = 2), miniature schnauzer (*n* = 2), Shih Tzu (*n* = 2), and one each of Pomeranian, Keeshond, Staffordshire terrier, Anatolian shepherd, husky, golden retriever, labradoodle, Great Dane, English bulldog, Dalmatian, Beagle, Weimaraner, and cocker spaniel. No specific breed was listed for one dog. The following diseases were represented: immune-mediated hemolytic anemia (IMHA; *n* = 9), immune-mediated thrombocytopenia (ITP; *n* = 4), immune-mediated polyarthritis (IMPA; *n* = 9), and other (*n* = 8). The other category consisted of two dogs with concurrent IMHA and ITP (Evan's-like syndrome), and one each of the following: immune-mediated pancytopenia, immune-mediated neutropenia, juvenile cellulitis, vasculitis with panniculitis, and non-specific immune-mediated cutaneous disease. Due to the timing of sample submission, four dogs had CXCL10 analyzed, but not vitamin D metabolites, thus providing 25 dogs for each analyte group. Nine healthy dogs comprised the CON group. The low number of healthy dogs in the study reflected budgetary considerations and the paucity of appropriate control dogs presented to the authors' tertiary referral teaching hospital.

### Vitamin D Metabolites

**Figure 1:**
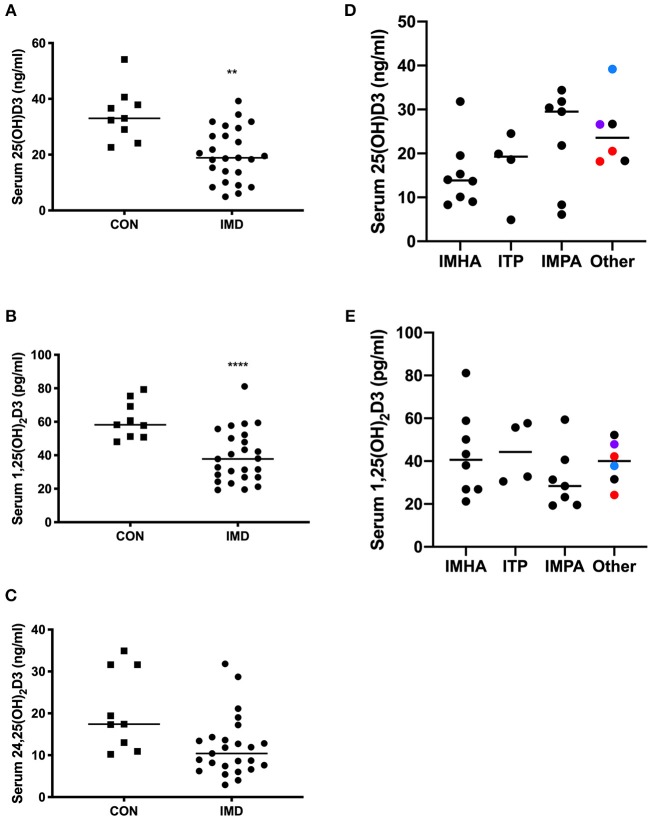
Serum concentrations of vitamin D metabolites in dogs (*n* = 25) with IMD and in health (*n* = 9). Dot plots compare 25(OH)D3 **(A)**, 1,25(OH)2D3 **(B)**, and 24,25(OH)2D3 **(C)** concentrations in IMD and CON dogs. Serum 25(OH)D3 **(D)** and 1,25(OH)2D3 **(E)** concentrations are compared between disease categories. Bars represent median values, and each dot corresponds to the value for an individual dog. Square plots represent control (CON) dogs, circles are dogs with immune-mediated disease (IMD). Colors of dots **(D,E)** correspond to the following, red = immune-mediated hemolytic anemia and thrombocytopenia combined, black = cutaneous/vasculitis disease, blue = pancytopenia, purple = immune-mediated neutropenia. ^**^P = 0.0018, ^****^P < 0.0001.

Serum 25(OH)D3, 1,25(OH)2D3, and 24,25(OH)2D3 was measured to determine the relationship between vitamin D and IMD in dogs. All dogs ate commercial diets (all reported diets adhered to adult maintenance requirements of the Association of American Feed Control Officers [AAFCO]) and hyporexia >1 week in duration was not reported. We compared serum concentrations between IMD and CON groups for each vitamin D metabolite. The concentrations of 25(OH)D3 (Figure 1A; *P* = 0.0018) and 1,25(OH)2D3 (Figure 1B; *P* < 0.0001) were 43 and 35% lower, respectively, in IMD vs. CON dogs. The median serum concentration of 24,25(OH)2D3 was not different (10.4 vs. 17.4 pg/ml, *P* = 0.0988) between IMD and CON dogs (Figure 1C).

Serum 25(OH)D3 (Figure 1D) and 1,25(OH)2D3 (Figure 1E) concentrations were compared between disease categories. Median concentrations of 25(OH)D3, while not significant (*P* = 0.093), differed the most (~2-fold) between dogs with IMHA and IMPA (Figure 1D).

### Serum CXCL10

**Figure 2:**
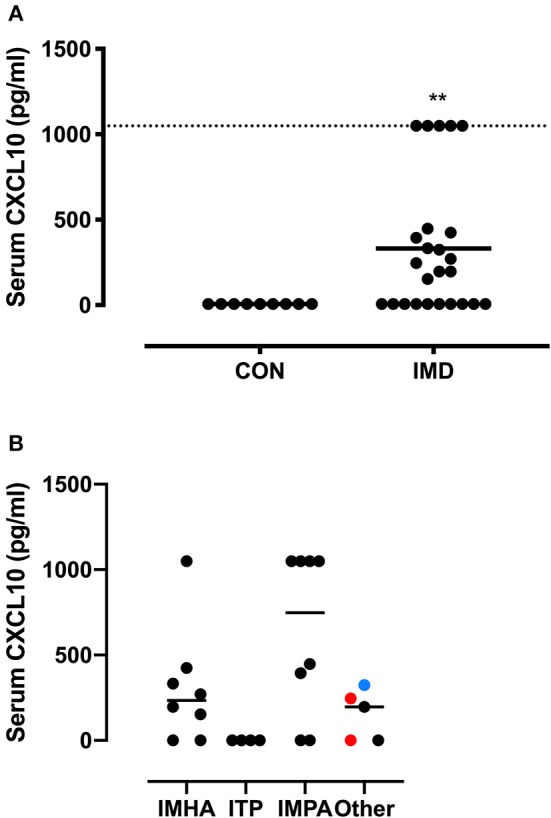
CXCL10 serum concentrations in dogs with IMD and healthy dogs. A dot plot compares serum CXCL10 concentrations between control (CON) and immune-mediated disease (IMD) dogs **(A)**. The dashed line indicates the upper range of quantification (>1,000 pg/ml). A dot plot compares serum CXCL10 concentrations between immune-mediated diseases **(B)**. Colors of dots **(B)** correspond to the following, red = immune-mediated hemolytic anemia and thrombocytopenia combined, black = cutaneous/vasculitis disease, blue = pancytopenia. ^**^P = 0.004.

As VDR activation suppresses CXCL10 gene expression, we measured the serum concentrations of this chemokine. Serum CXCL10 concentrations were below the sensitivity of the assay in all CON dogs (<6.8 pg/ml). The median serum CXCL10 concentration in dogs with IMD was 30 times greater (*P* = 0.004) than CON dogs (Figure 2A). CXCL10 concentrations did not correlate with 25(OH)D3 (*P* = 0.31) or 1,25(OH)2D3 (*P* = 0.41) concentrations. Although serum CXCL10 concentrations did not differ between disease categories (Figure 2B), 4/5 dogs with CXCL10 concentrations at the upper limit of quantification were diagnosed with IMPA.

### Patient Outcomes

**Figure 3:**
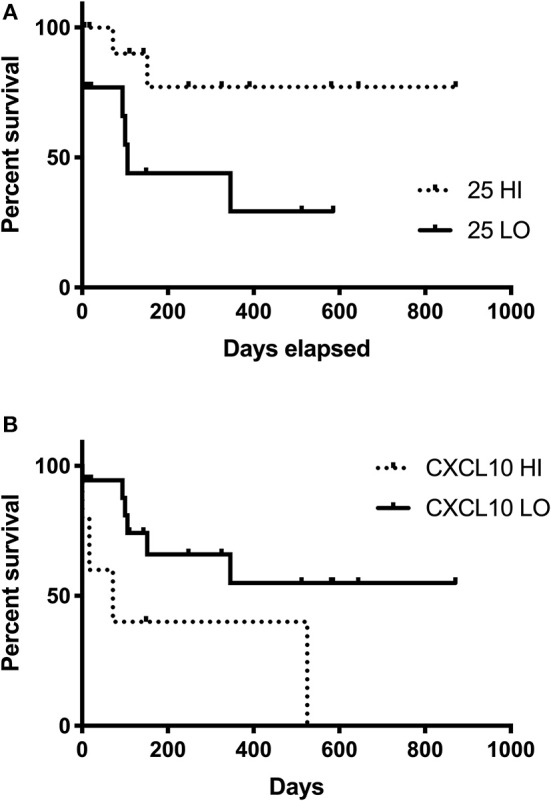
Survival time in dogs with IMD and relationship with serum 24(OH)D3 or CXCL10 concentrations. Survival curve for IMD dogs with 25(OH)D3 concentrations ≥ to the median (25-HI, dashed line) compared to IMD dogs with 25(OH)D3 concentrations < the median (25-LO, solid line) are significantly (*P* = 0.032) different **(A)**. Survival curve for the 5 IMD dogs with concentrations ≥1,000 pg/ml (CXCL10-HI, dashed line) and the remaining IMD dogs (CXCL10-LO, solid line) are significantly (*P* = 0.042) different **(B)**.

Patients with 25(OH)D3 concentrations ≥ the median (25-HI) survived longer than patients with 25(OH)D3 concentrations < the median (25-LO). Neither patient size (weight in kg) nor ages were different (*P* = 0.73, 0.15, respectively) between the 25-LO and 25-HI groups. While the 25-HI group did not reach a median survival time (MST; 77% survival at final censorship point), the MST was significantly (*P* = 0.032) shorter at 106 days in the 25-LO group (Figure 3A). Five dogs had serum CXCL10 concentrations ≥1,000 pg/ml (the upper limit of quantification). These five dogs (CXCL10 HI) survived 72 days, significantly (*P* = 0.049) shorter than the remaining IMD dogs (CXCL10 LO; Figure 3B). CXCL10 LO dogs did not reach an MST (71% survival at final censorship).

## Discussion

We demonstrate dogs, similar to other species, have reduced concentrations of serum vitamin D metabolites when afflicted with IMD. Autoimmune disease in people increases in incidence as populations further from the equator and corresponds with decreased ultraviolet mediated vitamin D synthesis. This geographic propensity is particularly true for multiple sclerosis (MS) where decreased vitamin D levels are associated with increased risk of disease. Clinical trials are currently investigating the benefit of vitamin D supplementation in MS patients (27–29). Experimental and naturally occurring autoimmune disease are treated with calcitriol and its analogs. Vitamin D analogs reduce the side-effect of hypercalcemia and in some cases enhance immune regulatory effects. Autoimmune disease models benefit from administration of 1,25(OH)2D3 or its analogs, including the non-obese diabetic mouse model and EAE (23, 30). Calcitriol and its analogs administered experimentally results in synergism with traditional immune suppressive agents such as cyclosporine A and mycophenolate mofetil (23).

Reduced vitamin D metabolite concentrations characterize various inflammatory and IMD. Vitamin D is reduced in dogs afflicted with spirocercosis (31) and congestive heart failure (32). In the case of congestive heart failure, reductions in vitamin D, within the reference range, were associated with shorter survival times (32). Interestingly, Vitamin D is reduced in canine patients with protein-losing enteropathy (PLE), but not with inflammatory bowel disease alone. This disparity is likely due to the impact on absorptive capacity and protein loss, including vitamin D binding protein in cases of PLE (33–35). One study measured lower vitamin D levels in cats with IBD and intestinal lymphoma (36). Chronic kidney disease can eventually result in lower plasma 1,25(OH)2D3 due to reductions in synthesis (34, 37). Thus, vitamin D status can be altered in cats and dogs secondary to inflammation, decreased absorption or synthesis and increased loss.

This study corroborates previous findings that dogs with certain IMD had significantly decreased 25(OH)D concentrations (38). To the authors' knowledge, this is the first study to compare Vitamin D levels with survival in dogs with IMD. Additional studies will be needed to determine if 25(OH)D3 levels correlate with controlling the course of immunologic disease.

Despite being the metabolite with the most biologic activity, 1,25(OH)D3 was not associated with survival times in patients with IMD. Calcidiol is the predominant and most stable vitamin D metabolite in circulation, and its levels reflect vitamin D status (39). Serum calcidiol can be converted directly into calcitriol by macrophages or other cells expressing 1-alpha-hydroxylase. Serum calcidiol concentrations likely better reflect systemic vitamin D availability. Calcitriol concentrations are likely more important at the tissue or cellular level, such as cases of IMD where lymphocytes and macrophages directly convert calcidiol to calcitriol.

That CXCL10 did not correlate with 1,25(OH)2D3 concentrations likely reflects complexity in induction and suppression of CXCL10 expression. Interferon gamma (IFNγ) stimulates CXCL10 expression (13), and therefore concentrations of IFNγ may override effects of VDR ligation. Additionally, numerous cell types including macrophages, T cells, fibroblasts, and endothelial cells synthesize CXCL10 (13), thus CXCL10 production may occur in cells, such as endothelial cells, that do not utilize 25(OH)D3 for autoregulation. Finally, the conversion rate of calcidiol to calcitriol locally may represent a rate-limiting step in regulating inflammation and IMD in which CXCL10 production continues unabated. Thus, immune cell microenvironments may be the only locations where a relationship exists between 25(OH)D3 and CXCL10 concentrations.

This study focused on vitamin D metabolite and CXCL10 concentrations at the time of diagnosis; however, longitudinal assessment of these parameters with treatment would answer questions regarding relationships between treatment responses and circulating vitamin D metabolites. As vitamin D is obtained only from the diet in dogs, dietary history is essential in any study involving canine vitamin D levels. All dogs in the study were eating commercial dog food diets and all diets reported in the medical record adhered to AAFCO standards. However, the lack of detailed feed analysis, the variability of vitamin D levels in commercial canine diets (40), and reductions in dietary intake that could not be accurately quantified may have impacted the results of this study. Measurement of vitamin D in the This study established a difference in vitamin D concentrations between healthy dogs and dogs with various IMD. We did not include an additional group of dogs with non-IMD illness for comparison as the purpose of this study was to determine if vitamin D concentrations are lower in dogs with IMD than in health. The authors recognize the importance of investigating the roles and relative contributions altered vitamin D concentrations may have in canine IMD compared to other disease processes as a future direction.

In conclusion, our data suggest that 25(OH)D3 and CXL10 are prognostic biomarkers in canine IMD. Assessments of serum 25(OH)D3 concentrations, separately by disease category and longitudinally with treatment, are warranted. Such studies may determine relationships between remission and vitamin D status that may differ for specific canine IMD. Low 25(OH)D3 or increased CXCL10 concentrations may be an indication for more aggressive treatment of IMD pending confirmation by further investigation. These data provoke consideration of studies that evaluate VDR agonists as an adjunctive therapy for specific canine IMD.

## Data Availability

All datasets generated for this study are included in the manuscript/supplementary files.

## Ethics Statement

The protocol was approved by the Cornell University Animal Care and Use Committee.

## Author Contributions

JL conceived and designed the study, performed the statistical analysis, and recruited cases and organized sample collection and storage. PM organized the data. JL and SP performed the assays. JL and PM wrote the first draft of the manuscript. JL, PM, and SP wrote sections of the manuscript. All authors contributed to manuscript revision, read, and approved the submitted version.

### Conflict of Interest Statement

The authors declare that the research was conducted in the absence of any commercial or financial relationships that could be construed as a potential conflict of interest.
